# Aortic Trauma Secondary to Automated Chest Compression Devices in the Setting of Out of Hospital Cardiac Arrest

**DOI:** 10.51894/001c.165683

**Published:** 2026-07-31

**Authors:** Andrew Loginsky, Alan Lazzara

**Affiliations:** 1 Henry Ford - Jackson

## 94

With no significant difference in return of spontaneous circulation (ROSC) in the setting of out of hospital cardiac arrest (OHCA) between those receiving manual chest compressions versus those receiving automated compressions from a device such as the Lund University Cardiopulmonary Assist System (LUCAS), it is important to recognize some of the more rare iatrogenic complications, such as aortic dissection/hematoma, associated with using the latter (1,4). We will explore a case of OHCA in a 62-year-old female with history of HTN, HLD, COPD and former tobacco use with approximately 29 minutes in arrest until ROSC was achieved, in which the LUCAS device was used, and is the most likely explanation of a Stanford type B aortic dissection/hematoma. She had been complaining for 2 days of progressive SOB and had cardiac arrest at home, at which time CPR was started by family members. Upon EMS arrival, a LMA was inserted and CPR resumed with the LUCAS device. The dissection was found incidentally on CT PE and was treated with anti-impulse therapy with vascular and cardiothoracic surgery on board. The patient has a poor prognosis attributed to anoxic brain injury, however with no difference in achieving ROSC, the result of an aortic hematoma/dissection should be recognized as a rare but serious complication of using a LUCAS device in OHCA.

